# Supplementary material for: A novel machine learning based framework for developing composite digital biomarkers of disease progression
Source: Front Digit Health. 2025 Jan 10;6:1500811. doi: 10.3389/fdgth.2024.1500811 (PMC11760596; doi:10.3389/fdgth.2024.1500811)
Supplement: Supplementary file 1 [file Datasheet1.pdf]

Supplementary Materials for

A Novel Machine Learning Based Framework for Developing Composite Digital Biomarkers of Disease Progression

Song Zhai[1], Andy Liaw[1], Judong Shen[1], Yuting Xu[1], Vladimir Svetnik[1], James J. FitzGerald[3, 4], Chrystalina A. Antoniades[3], Dan Holder[1], Marissa. F. Dockendorf[2], Jie Ren[2*], Richard Baumgartner[1*]

[1] Biostatistics and Research Decision Sciences, Merck & Co., Inc., Rahway, NJ 07065, USA

[2] Digital Clinical Measures, Translational Medicine, Merck & Co., Inc., Rahway, NJ 07065, USA

[3] NeuroMetrology Lab, Nuffield Department of Clinical Neurosciences, University of Oxford, Oxford, United Kingdom

[4] Nuffield Department of Surgical Sciences, University of Oxford, Oxford, United Kingdom

*Corresponding authors: Jie Ren (jie.ren@merck.com) and Richard Baumgartner (richard_baumgartner@merck.com)

Supplementary Materials:

Supplementary Method S1 – S2

Supplementary Figure S1 – S3

Supplementary Table S1 – S3

Supplementary Methods

Method S1. Penalized Generalized Estimating Equations (PGEE) for high-dimensional longitudinal data analysis

The Generalized Estimating Equations (GEE) approach has been widely applied to longitudinal data analysis. However, the direct use of traditional GEE in high-dimensional longitudinal data may lead to misleading results. In this analysis, we chose to use a ML-based Penalized GEE (PGEE) method, which performs simultaneous coefficient estimation and variable selection for longitudinal data analysis with high-dimensional variables (i.e., digital features). Specifically, the estimating function in GEE is defined as

$$S(\beta) = \frac{1}{N}\sum_{i=1}^{N} X_i^T V_i^{-1}(\beta)\left(Y_i - \mu_i(\beta)\right) = 0, \text{where } V_i(\beta) = A_i^{-1/2}(\beta)\, R\, A_i^{1/2}(\beta).$$

Here, $\beta = \left(\beta_1, \cdots, \beta_p\right)$ is the regression coefficients, and p is the number of digital features. N is the total number of subjects. R is a working correlation matrix. $X_i = \left(X_{i1}, \cdots, X_{in_i}\right)^T$ denotes the $n_i \times p$ matrix of covariates, and n_i is the number of visits for subject i. $A_i(\beta)$ is an $n_i \times n_i$ diagonal matrix with the marginal variance of responses.

We further add a penalty term to $S(\beta)$ in the PGEE estimating function:

$$U(\beta) = S(\beta) - q_\lambda(|\beta|) \circ \text{sign}(\beta), \text{where } q_\lambda(|\beta|) = \left(q_\lambda(|\beta_1|), \cdots, q_\lambda(|\beta_P|)\right)^T,$$

$$q_\lambda(t) = \lambda \times I(t < \lambda) + \frac{a\lambda - t}{a - 1} \times I(\lambda \le t < a\lambda) + 0 \times I(t \ge a\lambda).$$

Here, the tuning parameter λ determines the degree of shrinkage. Following the suggestion of Fan and Li (2001), we set $a = 3.7$. The notation $\circ$ denotes the component-wise product. And $q_\lambda(t)$ is the penalty function. Specifically, if $t < \lambda$, then $q_\lambda(t) = \lambda$,

indicating that the GEE $S(\beta)$ is fully penalized; on the other hand, if $t \geq a\lambda$, then $q_{\lambda}(t) = 0$, suggesting that the GEE $S(\beta)$ is not penalized.

Method S2. Algorithm of determining the optimal number of top features

Algorithm 1: Determine the optimal number of top features via cross-validation

Hyper-parameter: P

Initialization: Working correlation matrix R, penalty λ, K-fold CV

while $i \leq K$ **do**

> Rank all features (i.e., digital features) in terms of their PGEE estimates from the training set.
>
> Train different PGEE models with different number of top features (P) in the training set.
>
> Apply the PGEE models to the testing set, record the predicted outcomes of subjects in the testing set.

end

Calculate Root Mean Square Error (RMSE) or Area under the Curve (AUC) across all subjects with their observed and predicted outcomes, depending on the type of the endpoint. The optimal number of top features (P) is determined as the one with the smallest RMSE for the disease progression endpoint or the largest AUC for the disease status endpoint.

Output: P, RMSE or AUC

Supplementary Figures

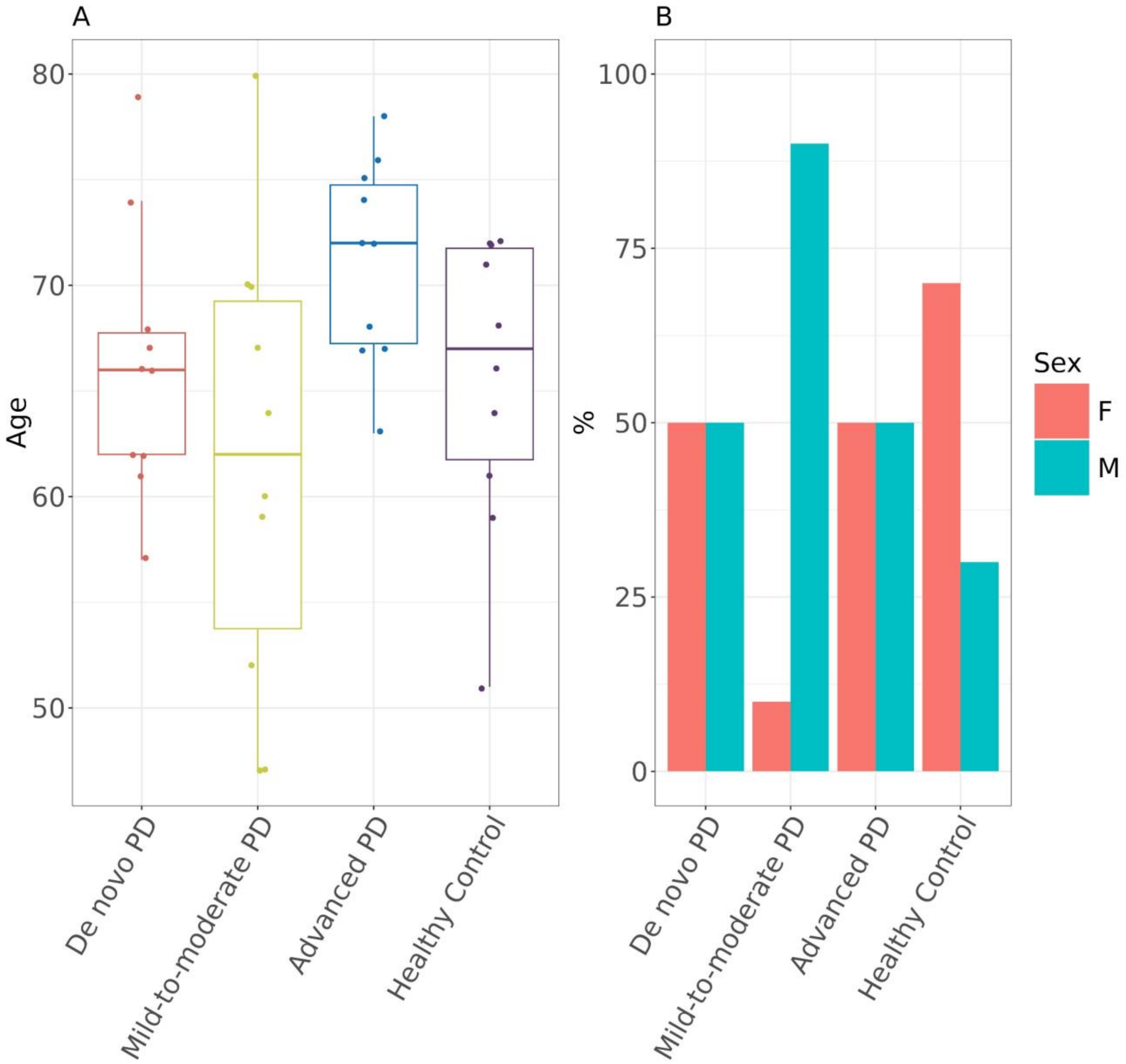

Figure S1. Distributions of age (A) and sex (B) in four different groups: de novo PD, mild-to-moderate, on-therapy PD, advanced PD, and HC.

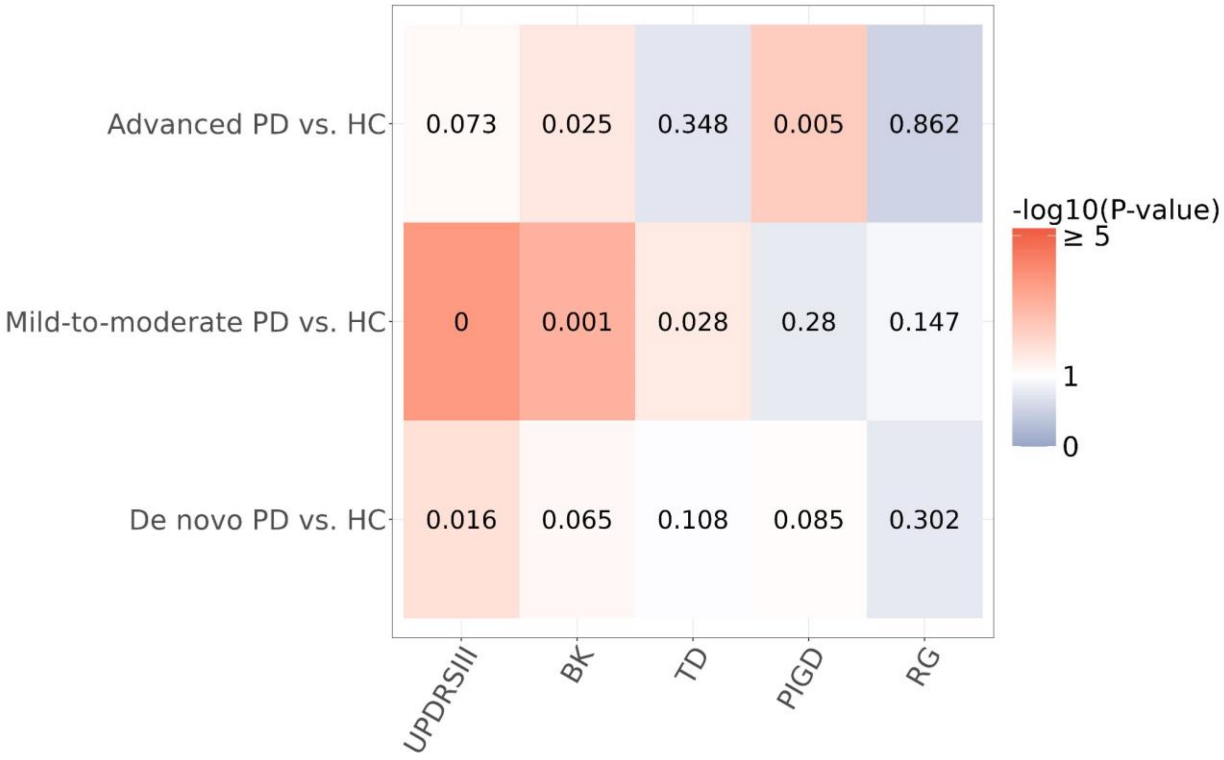

Figure S2. Heatmap of differential slope (i.e., progression) p-values between PD groups (i.e., de novo PD, mild-to-moderate PD, and advanced PD) and HC group using MDS-UPDRS Part III total score and its domain sub-scores. P-values were calculated from a linear mixed effects model with UPDRS III or its subscales (i.e., BK, TD, PIGD, and RG) as outcome variable. The independent variables included as fixed effects were intercept, group membership, age, visit, group-by-visit interaction, and age-by-visit interaction. Random intercept and slope were added as random effects. Original p-values were shown in the plot (with p-value threshold $\alpha = 0.1$).

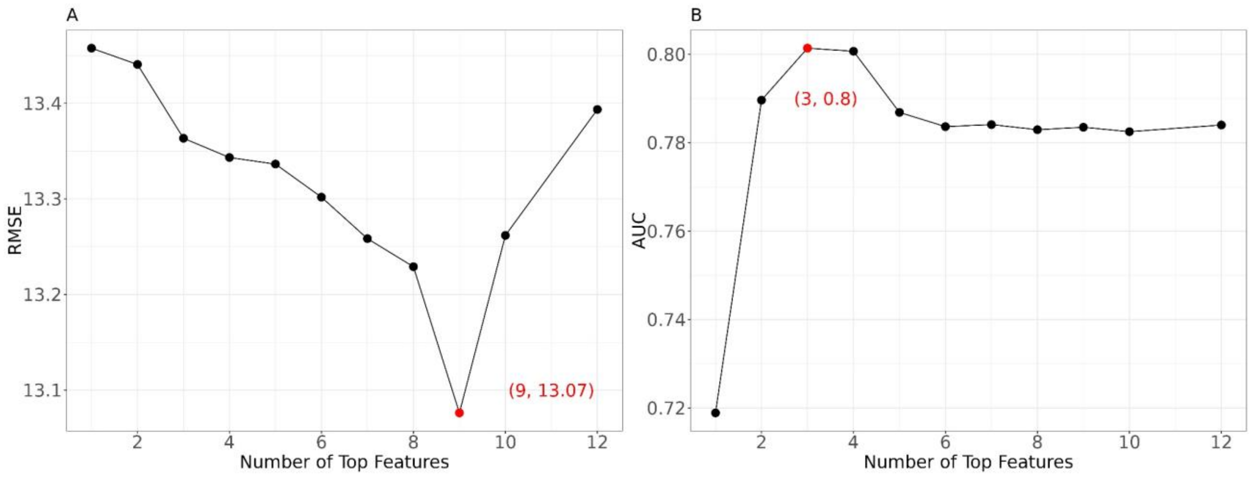

Figure S3. The optimal number of top features (i.e., digital features) to be selected into (a) MDS-UPDRS Part III prediction model, and (b) de novo PD versus HC classification model via a cross-validation procedure.

Supplementary Tables

Table S1. MDS-UPDRS Part III Subscales used in the analysis.

UPDRS Part III Subscale	Items from MDS-UPDRS Part III
Bradykinesia (BK)	3.13 Posture<br>3.14 Global spontaneity of movement (body bradykinesia)<br>3.4a Finger tapping LEFT HAND<br>3.4b Finger tapping RIGHT HAND<br>3.5a Hand movements LEFT HAND<br>3.5b Hand movements RIGHT HAND<br>3.6a Pronation supination LEFT HAND<br>3.6b Pronation supination RIGHT HAND<br>3.7a Toe tapping LEFT FOOT<br>3.7b Toe tapping RIGHT FOOT<br>3.8a Leg agility LEFT FOOT<br>3.8b Leg agility RIGHT FOOT<br>3.9 Arising from chair
Rigidity (RG)	3.3a Rigidity in NECK<br>3.3b Rigidity in LEFT ARM<br>3.3c Rigidity in RIGHT ARM<br>3.3d Rigidity in LEFT LEG<br>3.3e Rigidity in RIGHT LEG
Postural Instability and Gait Difficulty (PIGD)	3.10 Gait<br>3.11 Freezing of gait<br>3.12 Postural stability
Tremor dominant (TD)	3.15a Postural tremor LEFT HAND<br>3.15b Postural tremor RIGHT HAND<br>3.16a Kinetic tremor LEFT HAND<br>3.16b Kinetic tremor RIGHT HAND<br>3.17a Rest tremor LEFT ARM<br>3.17b Rest tremor LEFT LEG<br>3.17c Rest tremor RIGHT ARM<br>3.17d Rest tremor RIGHT LEG<br>3.17e Rest tremor LIP AND JAW<br>3.18 Constancy of rest tremor

Table S2. Determining whether sex and age (as covariates) would affect UPDRS III level at baseline and progression over time.

A. lme(UPDRS III ~ Sex*Visit, random = ~Visit|Subject ID, data = PD patients)			
Covariate	Estimate	SE	P-value
Sex	0.83	4.46	0.85
Visit	1.66	0.29	0.01
Sex:Visit	-0.66	0.50	0.19
B. lme(UPDRS III ~ Age*Visit, random = ~Visit|Subject ID, data = PD patients)			
Covariate	Estimate	SE	P-value
Age	0.26	0.24	0.30
Visit	-2.11	1.79	0.24
Age:Visit	0.05	0.03	0.04

Table S3. 77 Digital features that passed the univariate progression screening, including 15 postural sway features, 5 timed up-and-go features, and 57 two-minute walk features. P-values were calculated using linear mixed effects models.

Feature	P-value: group-by-visit	P-value: slope of de novo PD vs. HC	P-value: slope of mild-to-moderate PD vs. HC	P-value: slope of advanced PD vs. HC	P-value: slope of HC
Sway_PSAC95ER2:logMean	0.122	0.035	0.786	0.025	0.129
Sway_PSAC95ESA:logMean	0.040	0.006	0.204	0.009	0.187
Sway_PSACCMV:logMean	0.097	0.045	0.021	0.250	0.349
Sway_PSACCRMS:logMean	0.169	0.031	0.200	0.061	0.635
Sway_PSACMV:logMean	0.065	0.012	0.071	0.065	0.207
Sway_PSACRG:logMean	0.124	0.022	0.679	0.032	0.092
Sway_PSACRMS:logMean	0.141	0.037	0.771	0.031	0.144
Sway_PSACSMV:logMean	0.098	0.022	0.093	0.052	0.084
Sway_PSACSRG:logMean	0.103	0.028	0.991	0.032	0.067
Sway_PSACSRMS:logMean	0.112	0.031	0.673	0.019	0.126
Sway_PSAN95ER2:logMean	0.119	0.035	0.805	0.026	0.135
Sway_PSAN95ESA:logMean	0.040	0.006	0.199	0.009	0.189
Sway_PSANCRMS:logMean	0.165	0.031	0.192	0.061	0.639
Sway_PSANRMS:logMean	0.060	0.012	0.767	0.026	0.149
Sway_PSANSRMS:logMean	0.110	0.031	0.710	0.020	0.132
TUG_STSD:Mean	0.223	0.050	0.867	0.331	0.538
TUG_TA:logMAD	0.277	0.072	0.169	0.197	0.337
TUG_TD:logMAD	0.110	0.409	0.066	0.912	0.200
TUG_TD:Median	0.018	0.060	0.989	0.087	0.074
TUG_TPV:Median	0.008	0.001	0.147	0.013	0.780
Walk_APAMAA:logMean	0.179	0.031	0.549	0.267	0.947
Walk_GLLC:logMAD_A	0.052	0.600	0.474	0.018	0.568
Walk_GLLC:logMAD_L	0.007	0.708	0.524	0.012	0.922
Walk_GLLC:Median_A	0.006	0.002	0.430	0.037	0.369
Walk_GLLC:Median_L	0.005	0.002	0.439	0.038	0.362
Walk_GLLDS:logMAD_A	0.015	0.009	0.095	0.015	0.081
Walk_GLLDS:logMAD_L	0.002	0.026	0.009	0.001	0.146
Walk_GLLDS:Median_A	< 0.001	0.155	0.278	0.001	0.627
Walk_GLLDS:Median_L	< 0.001	0.191	0.288	0.002	0.663
Walk_GLLGCD:logSD_A	< 0.001	0.083	0.641	< 0.001	0.713

Walk_GLLGCD:logSD_L	0.002	0.082	0.949	0.001	0.881
Walk_GLLGCD:Median_A	0.012	0.004	0.408	0.050	0.292
Walk_GLLGCD:Median_L	0.008	0.003	0.408	0.042	0.294
Walk_GLLGS:logSD_A	0.042	0.015	0.330	0.002	0.055
Walk_GLLGS:logSD_L	0.164	0.032	0.509	0.021	0.072
Walk_GLLGS:Median_A	< 0.001	< 0.001	0.559	< 0.001	0.166
Walk_GLLGS:Median_L	< 0.001	0.002	0.425	< 0.001	0.240
Walk_GLLLSM:Median_L	0.133	0.529	0.345	0.045	0.327
Walk_GLLPIC:logMAD_L	0.073	0.059	0.610	0.068	0.279
Walk_GLLPIC:Median_A	0.011	0.020	0.362	0.008	0.485
Walk_GLLPIC:Median_L	0.146	0.071	0.454	0.087	0.519
Walk_GLLPTO:logMAD_A	0.009	0.048	0.137	0.001	0.837
Walk_GLLPTO:logMAD_L	< 0.001	0.002	0.083	< 0.001	0.297
Walk_GLLPTO:Median_A	0.002	0.069	0.843	0.002	0.507
Walk_GLLPTO:Median_L	< 0.001	0.226	0.404	< 0.001	0.212
Walk_GLLS:logMAD_A	0.132	0.490	0.618	0.025	0.887
Walk_GLLS:logMAD_L	0.002	0.014	0.626	0.006	0.260
Walk_GLLS:Median_A	0.038	0.389	0.848	0.020	0.506
Walk_GLLS:Median_L	< 0.001	0.288	0.026	< 0.001	0.444
Walk_GLLSD:logSD_A	< 0.001	0.007	0.409	< 0.001	0.915
Walk_GLLSD:logSD_L	0.002	0.088	0.486	0.001	0.769
Walk_GLLSD:Median_A	0.003	0.007	0.896	0.025	0.770
Walk_GLLSD:Median_L	0.118	0.027	0.222	0.151	0.169
Walk_GLLSLE:logSD_A	0.034	0.035	0.421	0.001	0.073
Walk_GLLSLE:logSD_L	0.040	0.004	0.441	0.022	0.078
Walk_GLLSLE:Median_A	< 0.001	0.015	0.746	< 0.001	0.197
Walk_GLLSLE:Median_L	< 0.001	0.029	0.493	< 0.001	0.258
Walk_GLLSLS:logMAD_A	0.275	0.075	0.362	0.222	0.390
Walk_GLLSLS:logMAD_L	0.251	0.327	0.194	0.056	0.921
Walk_GLLSLS:Median_A	< 0.001	0.239	0.047	0.001	0.551
Walk_GLLSLS:Median_L	0.034	0.298	0.786	0.017	0.461
Walk_GLLSW:logMAD_A	0.136	0.493	0.618	0.026	0.895
Walk_GLLSW:logMAD_L	0.002	0.014	0.631	0.006	0.261
Walk_GLLSW:Median_A	0.038	0.389	0.848	0.020	0.506
Walk_GLLSW:Median_L	< 0.001	0.289	0.026	< 0.001	0.445
Walk_GLLTDS:logMAD_A	0.012	0.057	0.102	0.002	0.362
Walk_GLLTDS:logMAD_L	< 0.001	0.005	0.009	< 0.001	0.940
Walk_GLLTDS:Median_A	< 0.001	0.208	0.179	< 0.001	0.391

Walk_GLLTDS:Median_L	0.004	0.253	0.631	0.010	0.497
Walk_GLLTOA:logMAD_A	0.018	0.714	0.304	0.012	0.974
Walk_GULMV:logMAD_A	0.069	0.250	0.970	0.059	0.410
Walk_GULMV:Median_A	< 0.001	0.129	0.793	0.003	0.224
Walk_GULMV:Median_L	0.027	0.312	0.770	0.015	0.432
Walk_GULROM:logMAD_L	0.086	0.147	0.389	0.458	0.742
Walk_TA:Median	0.181	0.265	0.055	0.194	0.274
Walk_TN:Mean	0.003	0.046	0.021	0.001	0.095
Walk_TPV:Median	0.085	0.221	0.370	0.018	0.659

References

Fan, J., and Li, R. 2001. Variable selection via nonconcave penalized likelihood and its oracle properties. *Journal of the American statistical Association 96* (456):1348-1360.
